# Latency of Infection

**Published:** 1896-01-11

**Authors:** 


The Hospital

An Institutional Journal of

The Medical Sciences and Hospital Administration, with

AN EXTRA NURSING SUPPLEMENT.

Vol. XIX.—No. 485.] [Jan. 11, 1896.

### Latency of Infection.

Whence come epidemics is a problem which still remains unsolved, notwithstanding the daily increasing store of information given us by bacteriologists. Nothing is simpler than to look on every step as due to infection, and to compare an epidemic to a prairie fire, in which the blaze, flashing from one clump of grass to another, sweeps across the plain; but the comparison is not true unless we bear in mind that it is only at certain times of year and in certain conditions of the herbage that prairie fires occur, and that these conditions are even more important factors in their production than the particular spark by which the conflagration is set up. Far too much is it the habit to consider the infectiousness of infectious diseases as the one predominant quality by virtue of which alone they spread. No doubt it is true enough that in a considerable number of cases it is possible to hazard a good guess as to the source from which infection has been derived; but it would be rash in the extreme to say that even the most careful search could discover it in all cases. It is well recognised that while many people are so insusceptible to certain diseases that they can be exposed to many chances of infection without evil results, others are so prone to them that an exposure so slight as not to be traceable, and certainly no greater than has been shared without effect by many others, is sufficient to give rise to the disease. Roughly, the causes of this individual susceptibility are spoken of as predisposing causes, and the exciting causes of the disease are considered to be exposure to an infection.

The recent discussion, however, at the Royal Medical and Chirurgical Society on the latency of parasitic germs puts the matter in a somewhat new light, showing that people may not only be infected, but many actually carry within them the germs of disease for considerable periods, until the onset of some accident, or some ailment quite unconnected with the infection, renders this effective in producing its proper series of symptoms. The infection thus becomes the predisposing condition, while the intercurrent malady acts as the exciting cause of the outbreak. The typical example of this sequence of events is to be found in cases in which tuberculous germs lie latent for years in a caseous mass without producing any general infection, when suddenly, as the result of some accident, the whole system becomes actively infected afresh and in a few weeks the patient dies from acute tuberculosis. Some remarkable cases also were related by Dr. Abraham in which patients who had only been in India when quite children developed leprosy many years afterwards; in one case soon after marriage, in the other after a febrile attack which was thought to be rheumatic fever.

More interesting still are the cases of typhoid fever mentioned by Sir William Broadbent, in which it seemed impossible to trace the origin of the infection during the normal period of incubation. It would seem as if the germ of that disease might at times lie latent, not doing any harm to the patient until brought into activity by some other cause, and Sir William Broadbent said that influenza had in the last five years so frequently ushered in typhoid that it seemed probable that the typhoid germ had been latent in the body, and had only been able to multiply when the resistance of the host was lowered by the onset of influenza. Such cases are in a somewhat different category from those of caseous tubercle, where the germs may be considered to be more or less encapsuled, and are more analogous with those mentioned by Dr. Washbourn, who stated that diphtheritic bacilli, pneumococci, streptococci, gonococci, and tubercle bacilli had been found on the mucous membrane of healthy individuals without producing any ill-effects. We need not enter into this matter further than to point out that while it is generally recognised that some people may throw off a degree of infection by which others would inevitably be laid low, a much more far-reaching principle is now propounded, namely, that people may, without either throwing the infection off or suffering from it, carry it about with them for varying periods, perchance infecting others, perchance under evil influences ultimately developing the disease themselves.

It is impossible to shut one's eyes to the important consequences which follow upon this view of affairs. The efforts of the sanitary authorities have for years been largely concentrated upon attempts to isolate those suffering from infectious diseases. This, so far as it goes, is very good, but the recent discussion does much to drive mere infection from its seat of pre-eminence in the causation of zymotic diseases, and to emphasise the over-ruling importance of those surrounding conditions on which it depends, whether or not this infection, be it active or latent, shall become effective. If people apparently healthy can go about carrying with them germs of disease, as we know is the case in regard to diphtheria, and as Koch has shown to be the case in cholera, the isolation of the actually sick can never be efficient in checking epidemics, and just as we find it necessary in regard to cholera to eliminate such causes as foul living, bad drainage, and polluted water supply, in addition to merely isolating the patients, so in regard to other infectious diseases we seem to have arrived at a point at which a study of their contributory causes, other than infection, may probably repay the community better than the mere perfecting of the machinery of isolation, useful as this is.

